# Female Urinary Incontinence: Frequency, Risk Factors, and Impact on the Quality of Life of Pregnant Pakistani Women

**DOI:** 10.12669/pjms.39.3.6313

**Published:** 2023

**Authors:** Saida Abrar, Raheela Mohsin, Anita Samad

**Affiliations:** 1 Dr. Saida Abrar, Assistant Professor OBGYN, Fellowship Urogynecology, Aga Khan University, Pakistan. Lady Reading Hospital, Peshawar, Pakistan; 2 Dr. Raheela Mohsin Rizvi, Clinical Fellowship Urogynecology, Sydney, Australia. Professor and Director, OBGYN, OMI Hospital, Karachi, Pakistan; 3 Dr. Anita Samad, Resident OBGYN, Aga Khan University Hospital, Karachi, Pakistan

**Keywords:** Urinary incontinence, Pregnancy, Prevalence, Risk factors

## Abstract

**Objectives:**

To determine the frequency and associated risk factors of urinary incontinence (UI), and its effect on the quality of life (QOL) of pregnant Pakistani women.

**Methods:**

This was a cross-sectional study of 309 pregnant women, 16-40 weeks gestation, age 18- 45 years, at the Aga Khan University Hospital Karachi, between August 2019 and February 2020. Data were obtained using the International Consultation on Incontinence Questionnaire-Urinary Incontinence-Short form (ICIQ-UI-SF).

**Results:**

The frequency of UI was 63.1%. Stress UI was the commonest type (53.0%) followed by urgency (17.5%) and mixed UI (11.7%). In majority of women, it occurred in small amounts, once a week with a very severe impact on QoL in 24.91% of women, affecting sexual relation the most. Risk factors for UI in pregnancy were age > 35 years (p < 0.02), gestation > 37 weeks (p< 0.00), higher body mass index and family history of UI (p< 0.00), previous instrumental vaginal delivery (P < 0.002), cough, constipation and strenuous job (p< 0.00), and lack of pelvic floor muscle exercises (p <0.03).

**Conclusions:**

UI is a common problem in pregnant women in Pakistan. It affects sexual functions the most, with a severe impact on the QOL, however, it commonly remains unreported. Thus the health care providers need to enquire all pregnant women on this issue, especially those at risk, and educate them on the available management options.

## INTRODUCTION

Urinary incontinence (UI) is defined by the International Continence Society (ICS), as ‘the complaint of any involuntary leakage of urine.^1^ The worldwide prevalence of UI in pregnancy is 41.0% (9–75%).^2^ About 423 million (21.6%) women suffered from UI in 2018, making it the greatest regional burden in Asia.^3^ The reported prevalence of UI in Pakistan is 11%^4^ and antenatal stress urinary incontinence (SUI) is 37%,^5^ which is characterized by involuntary leakage of urine on sneezing, coughing, or exertion. SUI is the commonest type in pregnancy and it increases with advancing gestational age.^6^ UI has a significant impact on both individuals and their families, and if left untreated, it gradually affects the quality of life (QOL) adversely.^7^,^8^

The pathophysiology of UI in pregnancy is multifactorial,^7^ including reduced bladder/urethral tone, weight gain, and urethral hypermobility.^9^ Currently, limited studies have been conducted in Pakistan to explore the prevalence of the condition. We aimed to determine the frequency of UI, its types, risk factors, and impact on QOL of pregnant women presenting at our hospital. This would estimate the burden of disease and would help us devise strategies to educate women for early reporting of their symptoms, and offer them rehabilitation for pelvic floor disorders, including UI during and after pregnancy.

## METHODS

This was a prospective, cross-sectional study of pregnant women at the Department of Obstetrics & Gynecology, Aga Khan University Hospital Karachi, between August 2019 and February 2020. Ethical approval was obtained from the Ethics and Research Committee (4950-Obs-ERC-17) in August 25, 2017.

A sample size of 309 was calculated using 5.3% the frequency of urgency UI (UUI), the margin of error 5%, and confidence interval at 95%.^10^ A non-probability consecutive sampling method was used.

It included all pregnant women aged 18-45 years, 16-40 weeks gestation (by last menstrual period), attended antenatal clinics at Aga khan University Hospital (AKUH) and who gave consent to participate in the study. All women with medical disorders of pregnancy like diabetes mellitus, hypertensive disorder of pregnancy, history of UI before pregnancy, or surgery for stress urinary incontinence (SUI) were excluded from the study. Participants were recruited from the Obstetrics clinics, and after taking informed consent, a structured proforma was used to collect data on socio-demographic and obstetric information like age, parity, gestational age, body mass index (BMI), occupation, educational status, and mode of previous deliveries. To assess the frequency of UI, its types, and its impact on QOL over the past 4 weeks, we used ICIQ-UI-SF developed by ICS.^11^ To get the ICIQ score, the individual scores of the frequency, amount of urinary leakage, and its interference with daily activities were added together. The ICIQ score has a range of 0 to 21, showing the severity of UI. ICIQ score 1-3 shows the light impact, 4-6 moderate impact, and 7-9 as severe impact; while 10 and above depicts very severe impact.

Data were analyzed using SPSS version 21. Qualitative variables were presented as frequencies and percentages, and means and standard deviations were used to summarize continuous variables. A P-value <0.05 was taken as significant. Body Mass Index (BMI) in kg/m^2^ was classified into four groups; underweight (≤19), healthy weight (19-24.9), overweight (25-29.9,) and obese (equal to or greater than 30).^12^ Chi-square, Fisher Exact, or independent t-test were used for bivariate analysis, where applicable.

## RESULTS

A total of 309 women were included in this study. The frequency of UI in this study was 63.1%. The mean age was 36.78±2.81 (Table-I). A total of 114 (36.9%) women were continent while 195 (63.1%) were incontinent. About 28.7% of the nulliparous and 27.7% of the multiparous women suffered from UI. SUI was the most common type of UI (56.9%) followed by UUI (18.77%) and mixed UI (MUI) (12.62%) respectively. Majority of the women reported leaking only a small amount of urine (82.05%), occurring once or less/ week (21.68%) (Table-II) and 88 (45.1%) did not report any lifestyle changes due to UI (Table-III). The sexual relationship was affected in more than half of the women. The mean ICIQ score was 12.11 ± 4.50, corresponding to a very severe effect on QOL, with a range of 3-21.

**Table-I:** Baseline demographics n =309.

Variable	Mean ± SD	Min-Max
Age (years)	36.78±2.81	20-40
Gestational age (weeks)	36.72±2.22	16-39
BMI (kg/m^2^)	29.72±1.56	27-34

BMI: Body Mass Index.

**Table-II:** Types/ Severity of Urinary Incontinence in Pregnancy n= 309

Variable	Number	Percentage (%)
***UI***		
No	114	36.9%
Yes Stress	195	63.1%
urinary incontinence	176	56.9%
Urgency urinary incontinence	58	18.77%
Mixed urinary incontinence	39	12.62%
***Episodes of UI***		
Never	114	36.90%
Once a week or less	67	21.68%
Twice or three times a week	48	15.53%
About once a day	37	11.97%
Few times a day	28	9.06%
Always	15	4.85%
***Amount of leak***		
Small	160	82.05%160/309=51.77%
Moderate	27	13.84%: 27/309=8.73%
Large	8	4.10%: 8/309= 2.5%

**UI:** Urinary incontinence.

**Table-III:** Impact on life of women with UI, n= 195.

***ICIQ-score***		
Continent	114	
Light impact	22	
Moderate impact	42	
Severe impact	54	
Very severe impact	77	
***Overall lifestyle changes by UI***		
None	88	45.1%
Shopping and excursions	10	10.2%
Outside home	9	4.6%
Working, performance, and friendship	08	4.1%
Daily home activities		
General health status	14	
Sexual relations	03	7.1%
Nervous and anxious	57	1.5%
Pad wearing and Protector	02	29.2%
	04	1.0%
		2.0%

ICIQ: International Consultation on Incontinence questionnaire.

Women with UI were significantly of higher age group, advanced gestation (>37 weeks) (p < 0.00, higher BMI (p< 0.00), had instrumental deliveries (p < 0.002), family history of UI (p< 0.00), constipation (p< 0.00), and cough (p< 0.00). Lack of Pelvic floor muscle exercises (PFME) was significantly associated with UI (p <0.03). The effect on educational status and parity was not statistically significant (Table-IV).

**Table-IV:** Comparison of factors between the continent and incontinent women in pregnancy n =309.

Variables	Incontinent(n=195)	Continent(n=114)	p-value
***Age group (years)***			0.02
< 35	44 (22.6%)	15 (13.2%)	
> 35	151 (77.4%)	99 (86.8%)	
***Gestational age (weeks)***			0.00
< 37	54 (27.6%)	82 (71.9%)	
> 37	141 (72.3%)	32 (28.1%)	
***BMI (kg/m^2^)***			0.00
Underweight	20 (10.2%)	49 (42.9%)	
Eutrophic	27 (13.8%)	28 (24.5%)	
Overweight	60 (30.7%)	24 (21.0%)	
Obese	88 (45.1%)	13 (11.4%)	
***Mode of the previous delivery***			0.00
Vaginal delivery	110 (56.4%)	62 (54.3%)	
Instrumental delivery	75 (38.4%)	08 (7.0%)	
Cesarean section	10 (5.1%)	44 (38.5%)	
***Parity***			0.36
Primipara	99 (50.76%)	64 (56.1%)	
Multipara	96 (49.2%)	50 (43.9%)	
***Educational status***			0.11
No formal education	11 (5.6%)	14 (12.3%)	
Primary education	57 (29.2%)	24 (21.1%)	
Secondary education	85 (43.6%)	49 (43%)	
Tertiary education	42 (21.5%)	27 (23.7%)	
***Occupational status***			0.00
Housewife	107 (54.9%)	83 (72.8%)	
Office job	57 (29.2%)	25 (21.9%)	
Strenuous job	31 (15.9%)	06 (5.3%)	
Family history of UI	35 (17.9%)	00 (00%)	0.00
Constipation	122 (62.6%)	48 (42.1%)	0.00
Cough	55 (28.2%)	04 (3.5%)	0.00
PFME	11 (5.6%)	2 (1.7%)	0.03
			

BMI: Body Mass Index, UI: Urinary incontinence, ICIQ: International Consultation on Incontinence Questionnaire, PFME: Pelvic floor muscle exercises.

## DISCUSSION

The current study showed the frequency of UI of 63.1% in pregnancy. This is higher than the reported prevalence of 40% of UI in East Asia, 43% in South Asia, 26% in Africa, and 36% in middle eastern populations, as shown in a study on multi-ethnic population of pregnant women in Norway.^13^ It is also much higher than the 37% prevalence of SUI shown in a local study of 520 pregnant patients in Liaquat University Hospital, Hyderabad.^5^ This reported low prevalence in Pakistan may be due to the under-reporting of UI in the rural areas, where women consider it taboo, hesitating to disclose their condition.

SUI was the commonest type of UI in pregnancy in the current study, which is in concordance with other studies.^14^ Obesity has been shown to double the risk of UI in pregnancy.^15^,^16^ Overweight and obesity were associated with UI in pregnancy in the current study also. The possible pathophysiology could be central obesity leading to increasing intra-abdominal pressure and hence increasing bladder pressure and urethral hypermobility.^17^ As revealed in other studies.^18,^ advanced gestation, strenuous physical activity, instrumental vaginal delivery, family history of UI, constipation, and cough also significantly increased the chances of developing UI in pregnancy.

Pelvic floor muscle exercises (PFME) are considered not only as of the most appropriate treatment for SUI during pregnancy but also to prevent future long-term complications like severe UI, fecal incontinence, and pelvic organ prolapse.^19^ However, the majority of women in our study were unaware of PFME and only 5.6% women in the continent group versus 1.7% in the incontinent group performed these exercises regularly.

Educational status and high parity were not risk factors for the development of UI in pregnancy. It is a well-established fact that pregnancy and childbirth cause damage to pelvic floor muscles, nerves, and connective tissues and can contribute to SUI.^20^ The higher prevalence among the nulliparous was unexpected and unlike the findings from other studies.^14^ This could be due to differences in the patients’ characteristics and health care services in our set-up exposing patients to a high risk of developing UI. Studies from Ethiopia and Turkey also made the same observations about parity, relating findings to the different health care systems and patients’ characteristics.^21^

Among women with symptomatic UI, most of them experienced leakage of a small amount, once a week, had mild symptoms, and did not make any lifestyle changes, similar to findings observed in a study from Nigeria.^22^ The mean ICIQ score revealed a very severe impact on QOL, unlike reported from a Nigerian study,^22^ but similar to a Brazilian study.^23^ In line with other studies, sexual relation was affected the most, especially in those who experienced moderate to severe symptoms.^24^

### Limitations:

Limitations of the study are its cross-sectional study design and thus inability to determine the temporal relationship to elicit the cause and effect of the disease. It is a single-centered study, so the findings cannot be representative of a larger population.

This is one of the few studies investigating the frequency of UI in pregnancy, its risk factors and its effect on QoL of women. These issues are rarely highlighted and studied in our country and as such the true burden of the problem and its impact on patient’s daily life are not addressed.

### Strength of the study:

Its strength lies in a large number of participants and it is important as to date, the prevalence of UI, its types, and the overall effect on QOL among pregnant women in Pakistan have not been widely investigated.

## CONCLUSION

In conclusion, UI in pregnancy is a common health problem in pregnancy, with SUI being the commonest type. It has severe effects on the QOL, affecting sexual relations the most. Despite this, the majority of the women do not volunteer these symptoms, either because of embarrassment or lack of knowledge about available treatment options. It poses a great challenge to our healthcare system and there is a dire need for more specialized units to increase our awareness, knowledge, and training in this area.

### Authors Contribution:

**SA:** Conceived, designed, data collection, and manuscript writing.

**RMR:** Did the editing and final approval of the manuscript. Responsible for integrity and accuracy of the work.

**SA:** Did the statistical analysis.

## References

[1] Haylen BT, de Ridder D, Freeman RM, Swift SE, Berghmans B, Lee J (2010). An International Urogynecological Association (IUGA)/International Continence Society (ICS) joint report on the terminology for female pelvic floor dysfunction. Int Urogynecol J.

[2] Moossdorff-Steinhauser HFA, Berghmans BCM, Spaanderman MEA, Bols EMJ (2021). Prevalence, incidence and bothersomeness of urinary incontinence in pregnancy:a systematic review and meta-analysis. Int Urogynecol J.

[3] Irwin Irwin DE, Kopp ZS, Agatep B, Milsom I, Abrams P (2011). Worldwide prevalence estimates of lower urinary tract symptoms, overactive bladder, urinary incontinence, and bladder outlet obstruction. BJU Int.

[4] Karim R, Begum S, Ayub S, Pervaiz KF, Akhtar R (2019). Incontinence of urine in pregnant women. J Postgrad Med Inst.

[5] Lata H, Qamar-ur-Nisa, Atta J, Goswami P (2011). The Pattern of Urinary Tract Symptoms During Pregnancy. Pak J Med Sci.

[6] Erbil N, Tas N, Uysal M, Kesgin A, Kilicarslan N, Gokkaya U (2011). Urinary incontinence among pregnant Turkish women. Pak J Med Sci.

[7] Jokhio AH, Rizvi RM, Rizvi J, MacArthur C (2013). Urinary incontinence in women in rural Pakistan:prevalence, severity, associated factors and impact on life. BJOG.

[8] Sacomori C, Cardoso FL (2015). Predictors of improvement in sexual function of women with urinary incontinence after treatment with pelvic floor exercises:A secondary analysis. J Sex Med.

[9] Shi Q, Wen L, Zhao B, Huang S, Liu D (2022). The Association of Hiatal Dimensions and Urethral Mobility With Stress Urinary Incontinence. J Ultrasound Med.

[10] Brown SJ, Donath S, MacArthur C, McDonald EA, Krastev AH (2010). Urinary incontinence in nulliparous women before and during pregnancy:Prevalence, incidence, and associated risk factors. Int Urogynecol J.

[11] Avery K, Donovan J, Peters TJ, Shaw C, Gotoh M, Abrams P (2004). ICIQ:A brief and robust measure for evaluating the symptoms and impact of urinary incontinence. Neurourol Urodyn.

[12] Okunola TO, Olubiyi OA, Omoya S, Rosiji B, Ajenifuja KO (2018). Prevalence and risk factors for urinary incontinence in pregnancy in Ikere-Ekiti, Nigeria. Neurourol Urodynam.

[13] BoK, Pauck Oglund G, Sletner L, Morkrid K, Jenum AK (2012). The prevalence of urinary incontinence in pregnancy among a multi-ethnic population resident in Norway. BJOG An Int J Obstet Gynaecol.

[14] Tingthong W, Buppasiri P, Chongsomchai C, Temtanakitpaisan T (2014). Prevalence of urinary incontinence in pregnant women at tertiary care hospitals in Khon Kaen province. Thai J Obstet Gynaecol.

[15] Townsend MK, Danforth KN, Rosner B, Curhan GC, Resnick NM, Grodstain F (2007). Body mass index, weight gain, and incident urinary incontinence in middle-aged women. Obstet Gynecol.

[16] Jaffar A, Mohd-Sidik S, Nien FC, Fu GQ, Talib NH (2020). Urinary incontinence and its association with pelvic floor muscle exercise among pregnant women attending a primary care clinic in Selangor, Malaysia. PLoS One.

[17] Fuselier A, Hanberry J, Margaret Lovin J, Gomelsky A (2018). Obesity and Stress Urinary Incontinence:Impact on Pathophysiology and Treatment. Curr Urol Rep.

[18] Akinlusi FM, Ottun TA, Oshodi YA, Seriki BO, Olalere FD, Kuye TO (2020). Female Urinary Incontinence:Prevalence, Risk Factors, and Impact on the Quality of Life of Gynecological Clinic Attendees in Lagos, Nigeria. Nepal J Obstet Gynaecol.

[19] Anderson KM, Davis K, Flynn BJ (2015). Urinary incontinence and pelvic organ prolapse. Med Clin North Am.

[20] Marinkovic SP, Rovner ES, Moldwin RM, Stanton SL, Gillen LM, Marinkovic CM (2012). The management of overactive bladder syndrome. BMJ.

[21] Bekele A, Adefris M, Demeke S (2016). Urinary incontinence among pregnant women, following antenatal care at University of Gondar Hospital, North West Ethiopia. BMC Preg Childbirth.

[22] Okunola TO, Olubiyi OA, Omoya S, Rosiji B, Ajenifuja KO (2018). Prevalence and risk factors for urinary incontinence in pregnancy in Ikere-Ekiti, Nigeria. Neurourol Urodynam.

[23] Oliveira C, Seleme M, Cansi PF, Consentino RF, Kumakura FY, Moreira GA (2013). Urinary incontinence in pregnant women and its relation with socio-demographic variables and quality of life. Rev Assoc Med Bras.

[24] Dolan LM, Walsh D, Hamilton S, Marshall K, Thompson K, Ashe RG (2004). A study of quality of life in primigravidae with urinary incontinence. Int Urogynecol J Pelvic Floor Dysfunc.

